# Taxonomic and conservation implications of population genetic admixture, mito-nuclear discordance, and male-biased dispersal of a large endangered snake, *Drymarchon couperi*

**DOI:** 10.1371/journal.pone.0214439

**Published:** 2019-03-26

**Authors:** Brian Folt, Javan Bauder, Stephen Spear, Dirk Stevenson, Michelle Hoffman, Jamie R. Oaks, Perry L. Wood, Christopher Jenkins, David A. Steen, Craig Guyer

**Affiliations:** 1 Department of Biological Sciences and Auburn University Museum of Natural History, Auburn University, Auburn, Alabama, United States of America; 2 The Orianne Society, 11 Fruitstand Lane, Tiger, Georgia, United States of America; 3 Department of Environmental Conservation, University of Massachusetts, Amherst, Massachusetts, United States of America; 4 Illinois Natural History Survey, University of Illinois, Champaign, Illinois, United States of America; 5 The Wilds, Cumberland, Ohio United States of America; 6 Altamaha Environmental Consulting, Hinesville, Georgia, United States of America; 7 The Orianne Center for Indigo Conservation, Central Florida Zoo and Botanical Gardens, Sanford, Florida, United States of America; 8 Georgia Sea Turtle Center, Jekyll Island Authority, Jekyll Island, Georgia, United States of America; University of Illinois at Urbana-Champaign, UNITED STATES

## Abstract

Accurate species delimitation and description are necessary to guide effective conservation of imperiled species, and this synergy is maximized when multiple data sources are used to delimit species. We illustrate this point by examining *Drymarchon couperi* (Eastern Indigo Snake), a large, federally-protected species in North America that was recently divided into two species based on gene sequence data from three loci and heuristic morphological assessment. Here, we re-evaluate the two-species hypothesis for *D*. *couperi* by evaluating both population genetic and gene sequence data. Our analyses of 14 microsatellite markers revealed 6–8 genetic population clusters with significant admixture, particularly across the contact zone between the two hypothesized species. Phylogenetic analyses of gene sequence data with maximum-likelihood methods suggested discordance between mitochondrial and nuclear markers and provided phylogenetic support for one species rather than two. For these reasons, we place *Drymarchon kolpobasileus* into synonymy with *D*. *couperi*. We suggest inconsistent patterns between mitochondrial and nuclear DNA are driven by high dispersal of males relative to females. We advocate for species delimitation exercises that evaluate admixture and gene flow in addition to phylogenetic analyses, particularly when the latter reveal monophyletic lineages. This is particularly important for taxa, such as squamates, that exhibit strong sex-biased dispersal. Problems associated with over-delimitation of species richness can become particularly acute for threatened and endangered species, because of high costs to conservation when taxonomy demands protection of more individual species than are supported by accumulating data.

## Introduction

Accurate species delimitation and description are critical not only for understanding global patterns of biodiversity, but also to guide effective conservation strategies [1–4]. For example, species are often delimited into multiple species on the basis of systematic studies utilizing molecular genetic data, thereby requiring adjustment of existing conservation management plans (e.g., [5]). When species delimitation methods fail to diagnose individuals (*sensu* [6]), such errors can have significant consequences for conservation and management of imperiled species by reducing or diverting finite conservation resources [2]. Therefore, taxonomic division into multiple species should be performed carefully and only when robust evidence supports a decision to revise. Indeed, some have cautioned that species delimitation studies should be conservative, because “it is better to fail to delimit species than it is to falsely delimit entities that do not represent actual evolutionary lineages” [7].

The process of species delimitation has greatly benefited from advancements in gene sequencing technology and the application of such data to infer phylogenetic trees and test hypotheses about species boundaries. Most recently, the field of molecular systematics has advanced by developing high-throughput sequencing technologies that measure genetic diversity across the entire genome [8]. These technologies provide tremendous data to estimate phylogenies, understand patterns of gene flow and admixture, and delimit species using thousands of genes (e.g., [9–11]). However, these technologies are not available to all researchers, particularly those working with non-model organisms, and numerous studies continue to delimit species by analyzing one or few genetic loci derived from Sanger-sequencing methods (e.g., [12–17]). This can be problematic because phylogenetic analyses of a single or few genetic loci frequently describe evolutionary patterns that do not reflect the organism’s true evolutionary history (i.e., the gene tree/species tree problem; [1,18–20]). In particular, use of and reliance on mitochondrial DNA (mtDNA) for phylogenetic and taxonomic analyses has been criticized because mtDNA has a vastly different natural history than the primary mode of genetic inheritance, nuclear DNA (nDNA). Compared to nDNA, mtDNA frequently is characterized by a lower effective population size, a higher mutation rate, and defying critical assumptions of neutral evolution by being under selection for vertebrates [4,21]. More importantly, mtDNA is maternally inherited and, therefore, may not describe an organism’s true patterns of inheritance expressed through the nuclear genome [21]. This is particularly problematic for species with relatively low dispersal rates that are more likely to show phylogeographic breaks that are not driven by decreased gene flow but by chance alone [22] and/or for species with intersexual differences in movement, site fidelity, or breeding behavior [23–25]. Given these limitations, research projects attempting to delimit species using a limited number of loci may greatly benefit from careful evaluation of alternative datasets, especially when focal species are of significant conservation concern [4].

In southeastern North America, *Drymarchon couperi* (Eastern Indigo Snake) is a large colubrid snake that was recently delimited into two species. First, Krysko et al. [26] used DNA sequence analyses to describe two genetic lineages of *D*. *couperi*–an Atlantic lineage, including populations in southeastern Georgia and eastern peninsular Florida, and a Gulf lineage of populations in western and southern peninsular Florida and the Florida panhandle. This phylogeographic study was followed by a second paper [16] that analyzed morphological variation between the Atlantic and Gulf lineages and provided an official description of the Gulf lineage as a novel species, *Drymarchon kolpobasileus* (Gulf Coast Indigo Snake). Because populations of *D*. *couperi* (*sensu lato*; hereafter referred to as *D*. *couperi*) have declined in abundance precipitously over the last century due to habitat loss, habitat fragmentation, and historical over-collecting for the pet trade [27,28], *D*. *couperi* is listed as Threatened under the U.S. Endangered Species Act [28,29]. Given this significant conservation designation, processes of species delimitation have extremely strong consequences for the conservation of Eastern Indigo Snakes. First, division of *D*. *couperi* into two smaller-ranged species results in two species with substantially smaller population sizes that are, therefore, at greater risk of extinction (*sensu* [2]; e.g., [30]). Second, conservation and recovery of two rare and imperiled species requires more time and funds than one species, and both resources are in short supply. Conservation assessments suggest that there are potentially viable populations of *D*. *couperi* remaining in large contiguous habitats in southeastern Georgia [31–33], and throughout peninsular Florida [33,34], but the species likely has been extirpated from Mississippi, Alabama, and the Florida panhandle [33]. In attempts to conserve the species from extinction, current conservation management plans for *D*. *couperi* were developed under the hypothesis that this binomial represents a single species. Additionally, as noted by Krysko et al. [26], active conservation management plans for *D*. *couperi* include repatriation projects in Alabama and western Florida [35], where populations attributed to the Gulf lineage were presumably extirpated but are being repatriated with genetic stock from the Atlantic lineage. Because repatriation projects should be informed by phylogeographic and systematic data [36], the description of *D*. *kolpobasileus* requires a renewed assessment of conservation status, captive breeding programs, and repatriation projects for *D*. *couperi*.

Despite recent taxonomic changes [16], important natural history and ecological data cause us to question the hypothesis that *D*. *couperi* comprises two independent evolutionary metapopulation lineages (i.e., species [37]). First, *D*. *couperi* movements can be extremely extensive, especially for males. Annual home range size for males can be as large as ca. 1500 ha [38] and average ca. 2.5–6.6 times larger than for females [38,39]. In fact, the disparity between male and female home range sizes becomes exacerbated in large snake species, a feature dominated by data from *D*. *couperi* (S1 Table). Within peninsular Florida, male *D*. *couperi* can move up to ca. 2 km in a single day and the average daily movement distance in males is approximately twice that of females [40]. Furthermore, males within peninsular Florida increase their movement frequency, distance, and home range size during the breeding season [40,41]. Dispersal distance of males may be 10 times that of females [42], and a small adult male in southern Georgia dispersed at least 22.2 km (straight line) over approximately two years [43]. Given the high movement and dispersal potential of *D*. *couperi*, it is hard to conceive that effective migration and gene flow between populations has not caused recent admixture and lineage reticulation among populations of *D*. *couperi*.

A second feature of *D*. *couperi* life history that reduces opportunities for speciation is the variety of habitats used by individuals throughout a year (e.g., [38]), particularly in peninsular Florida, where individuals will utilize habitats with varying degrees of anthropogenic disturbance [44]. Such diverse habitat use reduces the opportunity for ecological barriers to gene flow and, therefore, suggests potential for high admixture among populations. Additional life history observations show that *D*. *couperi* can cross freshwater and saltwater habitats 6–264 m wide [45] (D. Stevenson, personal observation; D. Breininger, unpublished data), and traditional river barriers [46] are thus unlikely to limit gene flow. Even if a historical climatic event separated *D*. *couperi* into two genetic populations [26], we hypothesize that high dispersal potential has resulted in recent admixture and gene flow among parapatric populations of *D*. *couperi*, thereby erasing historical population differentiation, a pattern also observed for diverse taxa following climatic cycles [47]. However, analysis of a multi-locus genetic dataset is needed to evaluate contemporary patterns of population structure and admixture for *D*. *couperi*.

To this end, we explored how incorporation of multi-locus population genetic data and natural history information could inform the existing model of species delimitation for *D*. *couperi*. We used a unified species concept [37,48] to define and operationally diagnose species of *D*. *couperi*, where species are necessarily defined as independently evolving metapopulation lineages and this feature must necessarily be demonstrated to delimit species [37]. To test the two-species hypothesis under the unified species concept, we analyzed a novel microsatellite DNA dataset and evaluated evidence of population admixture and gene flow between the two genetic lineages as an explicit test of whether there are two independently-evolving metapopulation lineages of *D*. *couperi*. We also re-examined published sequence data to test whether hypothesized lineages are supported by separate analyses of mtDNA and nDNA loci and whether phylogenetic inference is sensitive to different tree-generating algorithms. If *D*. *couperi* is two species, we made two predictions. First, we predicted that analyses of microsatellite data would describe two populations conforming to the Atlantic and Gulf lineages, with little or no admixture between the two, particularly at the putative contact zone identified by previous authors [16,26]. Second, we predicted that the hypothesized Atlantic and Gulf lineages would be supported by separate phylogenetic analyses of nuclear and mitochondrial data. By analyzing population genetic along with gene sequence data, we sought to provide a conceptually robust and integrative test [49,50] of whether *D*. *couperi* is two distinct species.

## Materials and methods

We extracted and genotyped microsatellite DNA from 428 tissue samples (scale, shed skins, or muscle from road-killed individuals) throughout peninsular Florida and southern Georgia. Twenty-five samples were obtained from the collections of the Florida Museum of Natural History, including 20 samples used in Krysko *et al*. [26]. The samples from Krysko *et al*. [26] included individuals from central Florida that represented both mitochondrial lineages where they occur in close proximity. The remaining Florida samples (N = 170) were collected during field studies of *D*. *couperi* [40,51] in and around Highlands County or opportunistically by authorized project partners. The samples from Georgia (N = 233) were collected by multiple project partners as part of a study of population fragmentation in the state (S. Spear et al., unpublished data). Our samples include similar representation of both mitochondrial lineages (55% Atlantic and 45% Gulf) as interpolated using maps from [26]. We extracted DNA using the Qiagen DNeasy blood and tissue extraction kit (Qiagen, Inc., Valencia, CA). We ran 17 microsatellite loci [52] within three multiplexed panels using the Qiagen Multiplex PCR kit (S2 Table for details). Each reaction contained 1X Qiagen Multiplex PCR Master Mix, 0.2 μM multiplexed primer mix (each primer at equal concentrations), and 1 μl of DNA extract in a total volume of 7 μl. The PCR protocol was modified from Shamblin et al. [52] for multiplex PCR and consisted of an initial denaturation of 95°C for 15 min, 20 touchdown cycles of 94°C for 30 s, 60°C minus 0.5°C per cycle for 90 s and 72°C for 1 min, followed by 30 cycles of 94°C for 30 s, 50°C for 90 s and 72°C for 1 min, and a final elongation step of 60°C for 30 min. Multiplexed PCR products were run on a 3130xl Applied Biosystems Genetic Analyzer at the University of Idaho’s Laboratory for Ecological, Evolutionary, and Conservation Genetics. We scored fragment sizes using Genemapper 3.7 (Applied Biosystems). We re-ran ~20% of samples to evaluate microsatellite error rates.

We tested for the presence of null alleles that would lead to violations of Hardy-Weinberg equilibrium assumptions using the software FreeNA [53] and excluded any loci that had an estimated null allele frequency > 0.10. We estimated population structure and number of genetic clusters using the Bayesian clustering algorithm Structure 2.3.4 [54]. We used the admixture model with 100,000 iterations following 10,000 burn-in repetitions. We evaluated *K* = 1–15 with 20 replicates for each value of *K* using the metrics proposed by Puechmaille [55]. We chose this method rather than the widespread Delta *K* method [56] to avoid issues associated with uneven sampling and because the Delta *K* method may be biased to describing support for *K* = 2 [55,57]. The Puechmaille [55] method calculates the proportion membership of each individual to each of *K* clusters; the mean and median of cluster membership is calculated across each sampling site, and the number of clusters that have a mean/median membership of a threshold (0.5 or greater) in at least one population is recorded. Therefore, the method only considers clusters that can be assigned to at least one sampling site. For each method (mean and median), the metric identifies two values of *K*: one that is the median value among all replicates, and one that is the maximum value among all replicates (i.e., four total estimates of *K*). Puechmaille [55] recommends calculating each of the four metrics at thresholds ranging from 0.5–0.8 to evaluate the most consistent number of clusters. Our sampling did not generally fit into discrete geographic clusters; therefore, we used county to define sites. Although counties are not biological entities, they should still be appropriate for calculating the four metrics given the assumption that there would not be population subdivision within counties. Given the extensive home ranges of *D*. *couperi*, we believe this assumption is reasonable. We used the median value of *K* from the 16 different possibilities (four types of metrics multiplied by four different thresholds). We estimated the Puechmaille [55] metrics using StructureSelector [58]. We tested for evidence of population differentiation in the 20 samples used by Krysko *et al*. [26] that represented spatial overlap of Atlantic and Gulf lineages by estimating Jost’s D metric of genetic differentiation [59]. Jost’s D was developed to better represent actual levels of genetic differentiation when markers with high mutation rates (such as microsatellites) are used. We estimated Jost’s D using the ‘mmod’ package [60] in R.

We conducted two spatial analyses to test for the presence of genetic structure while accounting for spatial autocorrelation. First, we estimated the number of genetic clusters using the spatial Bayesian clustering algorithm Geneland v. 4.0.8 [61] in R using uncorrelated allele frequencies and filtering null alleles. We ran 10 independent runs using 5,000,000 iterations and saved every 100^th^ iteration for post-processing, discarding the first 25% of these as burn-in. We tested *K* = 1–12, varied the number of populations along the Markov chain Monte Carlo (MCMC) inference, set the maximum rate of the Poisson process equal to the number of samples, and did not assign spatial uncertainty to the spatial coordinates of our samples. We made inferences from the run with the highest mean posterior and assigned samples to the cluster with the highest proportional probability of membership. We also conducted a spatial principle components analysis (sPCA) to identify spatial patterns of genetic structure while accounting for spatial autocorrelation among samples without relying on assumptions of Hardy-Weinberg equilibrium [62]. sPCA requires specifying a connection network to define connected samples. We evaluated three connection networks: (1) Delaunay triangulation, (2) Gabriel graph, and (3) a distance-based connection network, where samples ≤ 22.2 km (the maximum known dispersal distance by Eastern Indigo Snakes; [43]) were considered connected. We conducted significance tests for global and local structure using 9,999 permutations in the package ‘adegenet’ [63] in R.

We analyzed the microsatellite data using linear mixed-effects models with maximum-likelihood population effects (MLPE; [64]) to better understand the role of isolation by distance in explaining genetic distance within and among lineages. We estimated genetic distance at the individual level using 1—proportion of shared alleles [65], where increased values indicated greater genetic dissimilarity between samples. To test for isolation by distance, we built three MLPE models examining how genetic distance varied by Euclidean geographic distance: a model using only Atlantic lineage samples, a model using only Gulf lineage samples, and a model using all sample from both lineages. For each model, we report the parameter estimate for Euclidean distance, it’s standard error and *t* statistic, and the marginal *R*^*2*^ (i.e., the proportion of variance explained by fixed-effect factors; [66,67]). To visualize how isolation by distance differed within and between lineages, we graphically overlaid genetic distance against Euclidean distance for within-lineage distances against genetic distances measured between lineages to see how isolation by distance varied within lineages relative to across all samples. If mitochondrial lineages represented different species, we predicted lower genetic distances within lineages than between lineages (i.e., no or little overlap in genetic distances within and among lineages). We estimated proportion of shared alleles using the package ‘adegenet’ [63] and implemented MLPE tests with individuals as random effects using the package ‘ResistanceGA’ [68] in R.

To infer evolutionary history among populations of *D*. *couperi*, Krysko *et al*. [26] analyzed sequence data obtained from three genetic markers: two linked mtDNA genes (cytochrome *b* [CytB], nicotinamide adenine dinucleotide dehydrogenase subunit 4 [ND4] and two adjacent non-coding tRNAs [Histidine and Serine]), and one nuclear gene (*neurotrophin*-3 [NT3]). The mtDNA was sequenced for 72 specimens, while the nDNA was sequenced for a subset of 23 specimens. The authors estimated phylogenetic relationships among populations by analyzing a concatenated dataset including both mitochondrial and nuclear loci using maximum likelihood (ML) and Bayesian inference (BI) analyses. Because both analyses generated similar phylogenetic hypotheses, the authors described results from only the concatenated Bayesian analysis. However, a customary practice in phylogenetic studies is to use both mitochondrial and nuclear loci and to describe phylogenetic patterns inferred from these two components of the genome separately (e.g., [19,69–71]). This practice can help identify situations for which phylogenetic hypotheses generated from mtDNA (1) are incongruent with hypotheses from the nuclear genome and that (2) might be erroneously assumed to accurately depict the species tree. However, because Krysko *et al*. [26] combined the mitochondrial and nuclear markers and used that concatenated dataset to infer both ML and Bayesian phylogenies from the combined datasets, their sequence analyses may be biased toward describing patterns from maternally inherited mtDNA.

To explore the extent to which mitochondrial and nuclear sequence data separately support lineage divergence within Eastern Indigo Snakes, we accessed the available sequence data from GenBank using the accession numbers listed in [26] (S3 Table) and used ML methods to infer separate mitochondrial and nuclear gene trees, largely following the methods used by Krysko *et al*. [26]. The mitochondrial data (cytochrome *b*, ND4 and non-coding tRNAs Histidine and Serine) included 72 individuals of *D*. *couperi* (N = 28 Atlantic lineage; N = 44 Gulf lineage) and the nuclear data included 23 individuals (N = 13 Atlantic lineage, N = 10 Gulf lineage); both datasets had five outgroup samples (*Drymarchon melanurus erebennus*, *Drymarchon melanurus rubidus*, *Coluber constrictor*, *Masticophis flagellum*, and *Salvadora mexicana*). We fit different models of nucleotide substitution, ranked them using Bayesian Information Criterion (BIC), and inferred phylogenetic trees using IQTREE v. 1.6.4 ([72]) on the Mary Grace Hopper super-computer cluster at Auburn University. We partitioned the mitochondrial data by coding and non-coding regions [73]. We evaluated node support using ultrafast bootstrapping with 1000 replicates [74]. Because our ML analyses inferred different phylogenetic structure than that of [16,26], we additionally analyzed the mtDNA with BI methods approximating the methods of [26]. However, some details needed to reproduce these analyses were unclear (e.g., tree prior) so we conducted our BI analysis as follows. We ran BI analyses in BEAST v2.5.1 [75] using the partitioned mtDNA under different tree priors. We applied both a Yule tree prior and an Extended Bayesian Skyline Plot (EBSP) prior and performed MCMC for 100 million generations sampling every 10,000 generations. We implemented models of molecular evolution following [26] and also applied model averaging using bModelTest [76]. We visualized our log files using Tracer v1.5 [75] to ensure the MCMC chains reached stationarity and that the effective samples sizes (ESS) of each parameter were greater than 200. Lastly, we used TreeAnnotator v2.4.6 [77] with a 25% burn-in to generate maximum clade credibility (MCC) trees. We did not analyze the nuclear locus using BI methods because during the ML analyses we learned that the locus was invariant among *D*. *couperi* specimens.

The use of live snakes for research was approved by Auburn University IACUC protocols (PRN 2007–1142, 2010–1750, 2013–2386, 2017–3102) and a federal research permit from the United States Fish and Wildlife Service (TE32397A-O). All DNA samples used to generate the microsatellite data are accessioned in the Auburn University Museum of Natural History or the Florida Museum of Natural History; our microsatellite data and accession numbers for the DNA samples are available on the digital data repository ‘figshare’ (https://doi.org/10.6084/m9.figshare.7637729.v1). Gene sequence data are available on GenBank with accession numbers from S3 Table [26].

## Results

**Fig 1:**
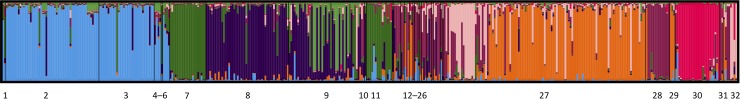
Bar plots of population clustering estimated through the Bayesian clustering algorithm Structure with *K* = 8 for specimens of *Drymarchon couperi* (Eastern Indigo Snake). The y-axis is the proportion of individual ancestry for each cluster; in the x-axis, number represents county where sample was collected. County names for each number are shown in S5 Table. Some counties (e.g., 12–26) are overlapping on the x-axis due to small sample size per cluster.

**Fig 2:**
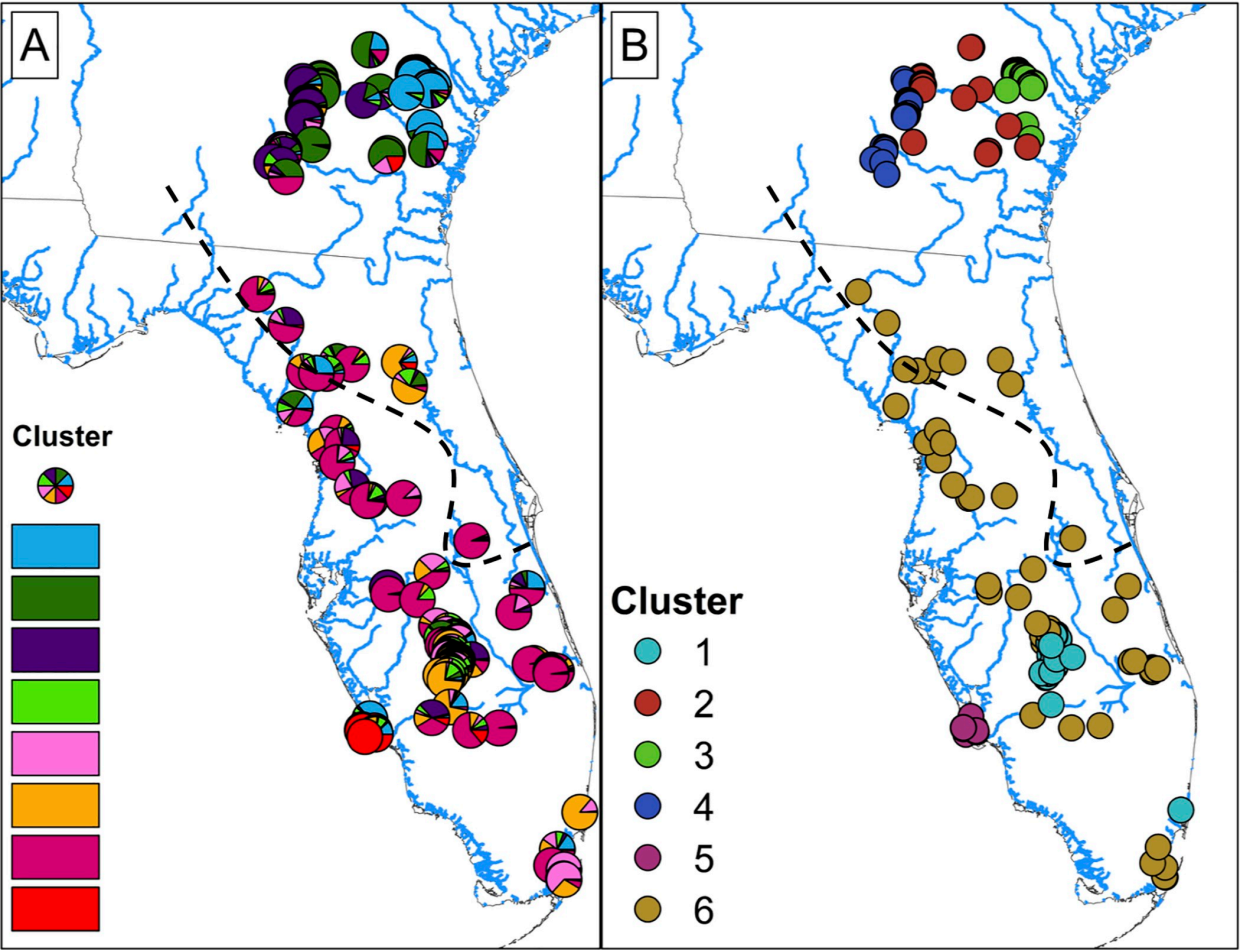
Maps of *Drymarchon couperi* (*sensu* lato) sampling sites represented as (A) pie charts of percent ancestry within population clusters identified by Structure analyses with *K* = 8 populations, and (B) cluster membership from the Geneland analysis with *K* = 6 populations. For both panels, percent ancestry and/or cluster membership was assigned given the number of populations *K* that received the highest support during analyses. The black dashed lines indicates the boundary between the Atlantic and Gulf lineages from [26]. For panel (A), colors are as in Fig 1.

We found evidence for null alleles in three loci (Dry33, Dry63, and Dry69). Therefore, we eliminated these loci from further analysis and retained the remaining 14 loci. We estimated an error rate of 0.05 while re-running samples. Values of *K* estimated by Structure ranged from 6–10 depending on metric and threshold (S4 Table); because *K* = 8 was the median value across all 16 combinations, we use that for inference (Fig 1). There was extensive admixture among clusters inferred by Structure (Fig 1) and Geneland (Fig 2), especially in central Florida at the contact zone between the two hypothesized species. For instance, the maximum ancestry value from Structure for any one cluster for central Florida was only 0.61, compared to an average value of 0.80 for individuals from Georgia. The most differentiated genetic clusters were primarily in the northern extent of the range in Georgia with an additional highly differentiated population associated with Gulf islands of Lee County, Florida (Fig 2). Samples from the putative contact zone had an extremely small Jost’s D value (0.0004), further indicating no genetic differentiation among samples at the putative contact zone between the two species. Structure plots describing different values of *K* are reported in SI S1 Fig.

Geneland identified clear support for *K* = 6 (SI S2 Fig) across all 10 independent runs. Within each run, all Georgia samples were contained exclusively within three clusters, while Florida samples were contained exclusively within the remaining three clusters. The highest mean posterior was 136.20 units higher than the second highest mean posterior. Within the highest-ranked run, all Atlantic lineage samples within peninsular Florida were within the cluster that also included Gulf lineage samples from across peninsular Florida (Fig 2).

**Fig 3:**
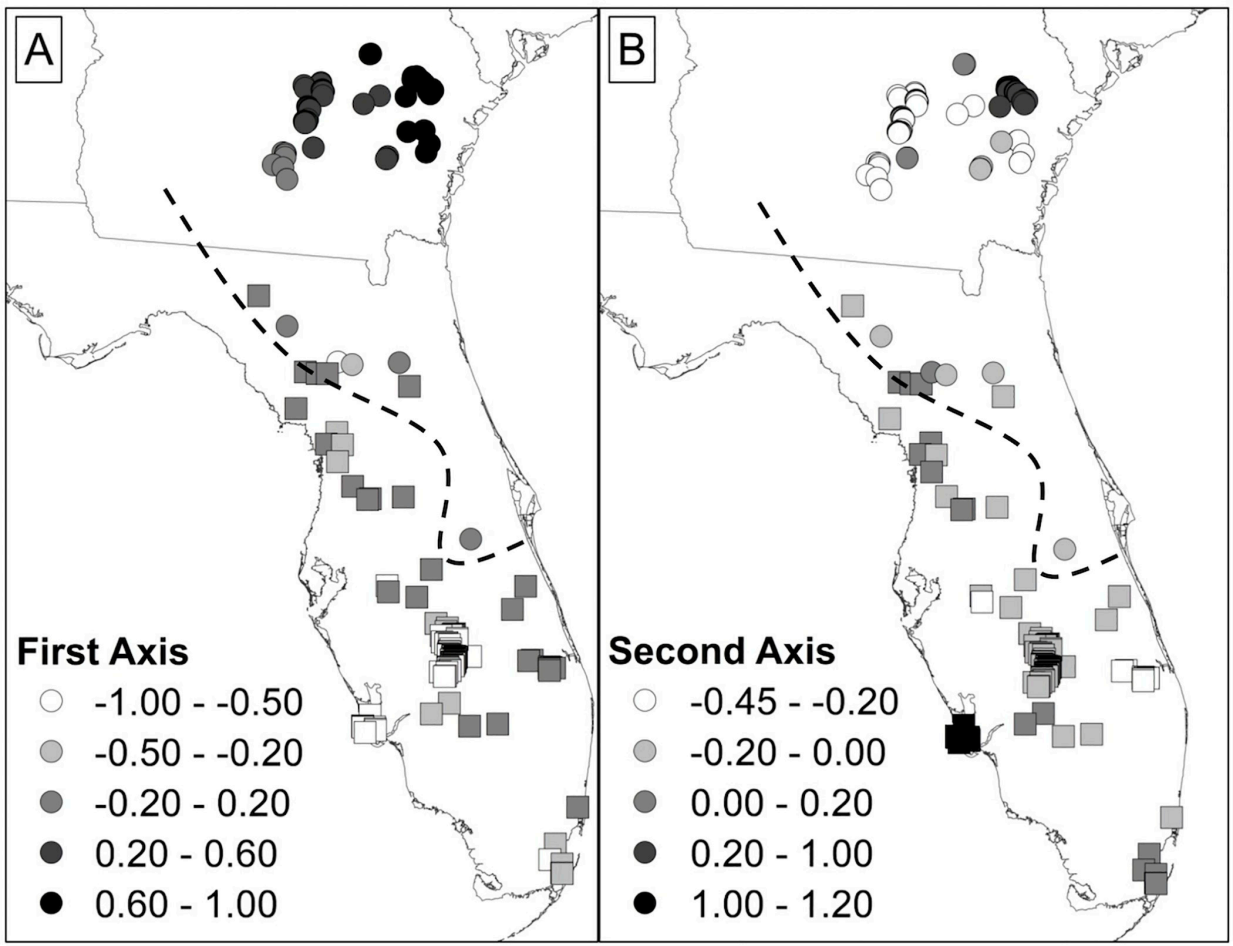
Spatially lagged scores for each sample from the first (A) and second (B) axes of a spatial principle components analysis (sPCA). Samples with more extreme values/colors are more genetically differentiated. Atlantic lineage samples [26] are displayed using circles while Gulf lineage samples are displayed using squares; the black dashed lines indicates the boundary between the Atlantic and Gulf lineages.

Inferences about spatial genetic structure using sPCA were very similar among the three connection networks we used, so we report the results using the distance-based connection network (see SI S3 and S4 Figs for results using the Delaunay triangulation network). Lagged scores from the first two PC axes were highly correlated between different connection networks (*r* ≥ 0.93). The global test was significant (observed = 0.0222, P < 0.0001) while the local test was not (observed = 0.0064, P = 0.84). The first PC axis explained the most variation followed by the second PC axis (0.2978 and 0.1533, respectively; all other axes ≤ 0.1002; SI S5 Fig) and both axes showed positive spatial autocorrelation (Moran’s *I* = 0.83 and 0.74, respectively; SI S5 Fig). The lagged scores of the first axis suggested genetic structure follows a north-south gradient, while the second axis suggested strongest genetic structure within southern Georgia and between Georgia and Florida (Fig 3). There was no consistent differentiation between samples from different lineages and relatively substantial overlap across the putative contact zone.

**Fig 4:**
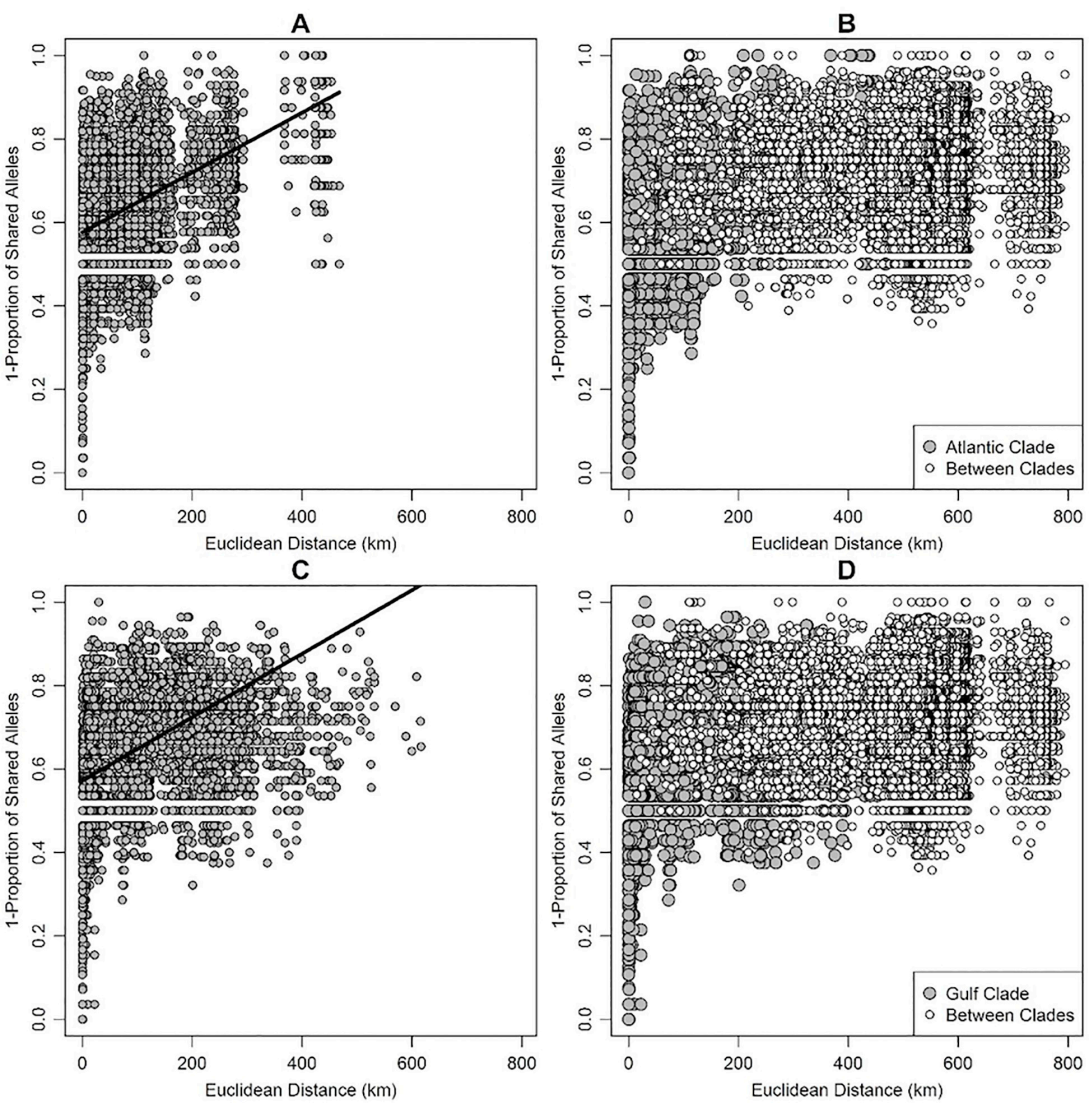
Plots of pairwise genetic distance (1—proportion of shared alleles) against Euclidean distance (km) showing positive isolation by distance. Solid lines show the predicted pattern of isolation by distance from a linear mixed-effects model with maximum-likelihood population effects (MLPEs; [64]). (A) Pairwise distances within Atlantic lineage samples; (B) pairwise distances within Atlantic lineage samples (gray circles) and among samples from both Atlantic and Gulf lineages (white circles); (C) pairwise distances within Gulf lineage samples; (D) pairwise distances within Gulf lineage samples (gray circles) and among samples from both Atlantic and Gulf lineages (white circles).

MLPE models described strong effects of Euclidean distance on genetic distance among samples. Specifically, we observed significant effects of Euclidean distance on genetic distance within the Atlantic lineage (β = 0.05, SE = 0.0007, *t* = 65.5, *R*^*2*^ = 0.21), the Gulf lineage (β = 0.07, SE = 0.001, *t* = 48.6, *R*^*2*^ = 0.34), and among all samples (β = 0.04, SE = 0.0003, *t* = 148.8, *R*^*2*^ = 0.18). When comparing genetic distance within and between mitochondrial lineages, values within lineages overlapped greatly with values observed among all samples between lineages (Fig 4).

**Fig 5:**
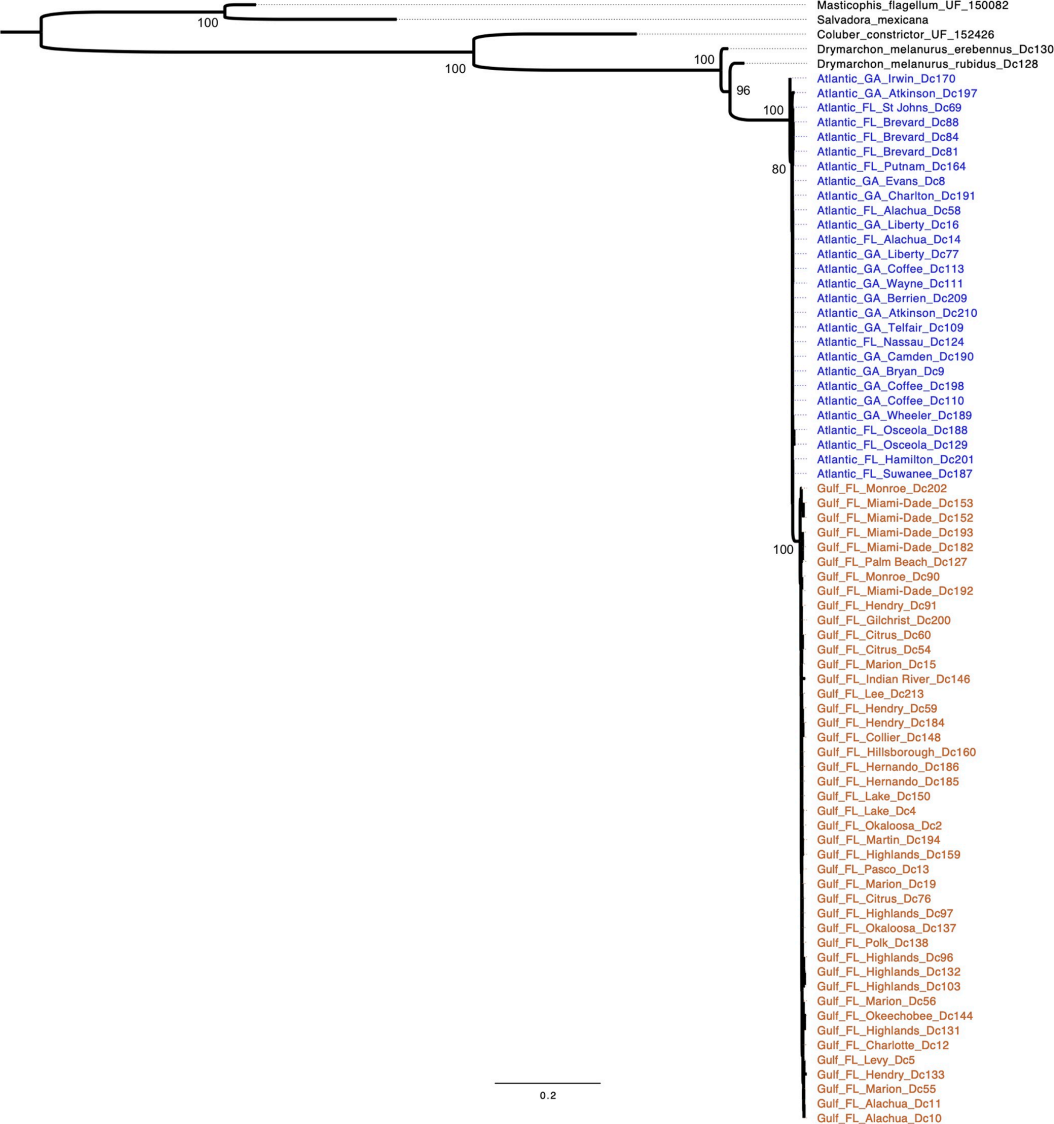
Maximum-likelihood phylogeny of Eastern Indigo Snakes (*Drymarchon couperi*) and outgroups inferred from sequence data from the mitochondrial loci cytochrome *b* and ND4. Indigo snake samples are labeled by hypothetical lineage, state, county, and sample numbers from [26]. Colors: blue = Atlantic lineage, orange = Gulf lineage. Bootstrap support is listed for major nodes.

**Fig 6:**
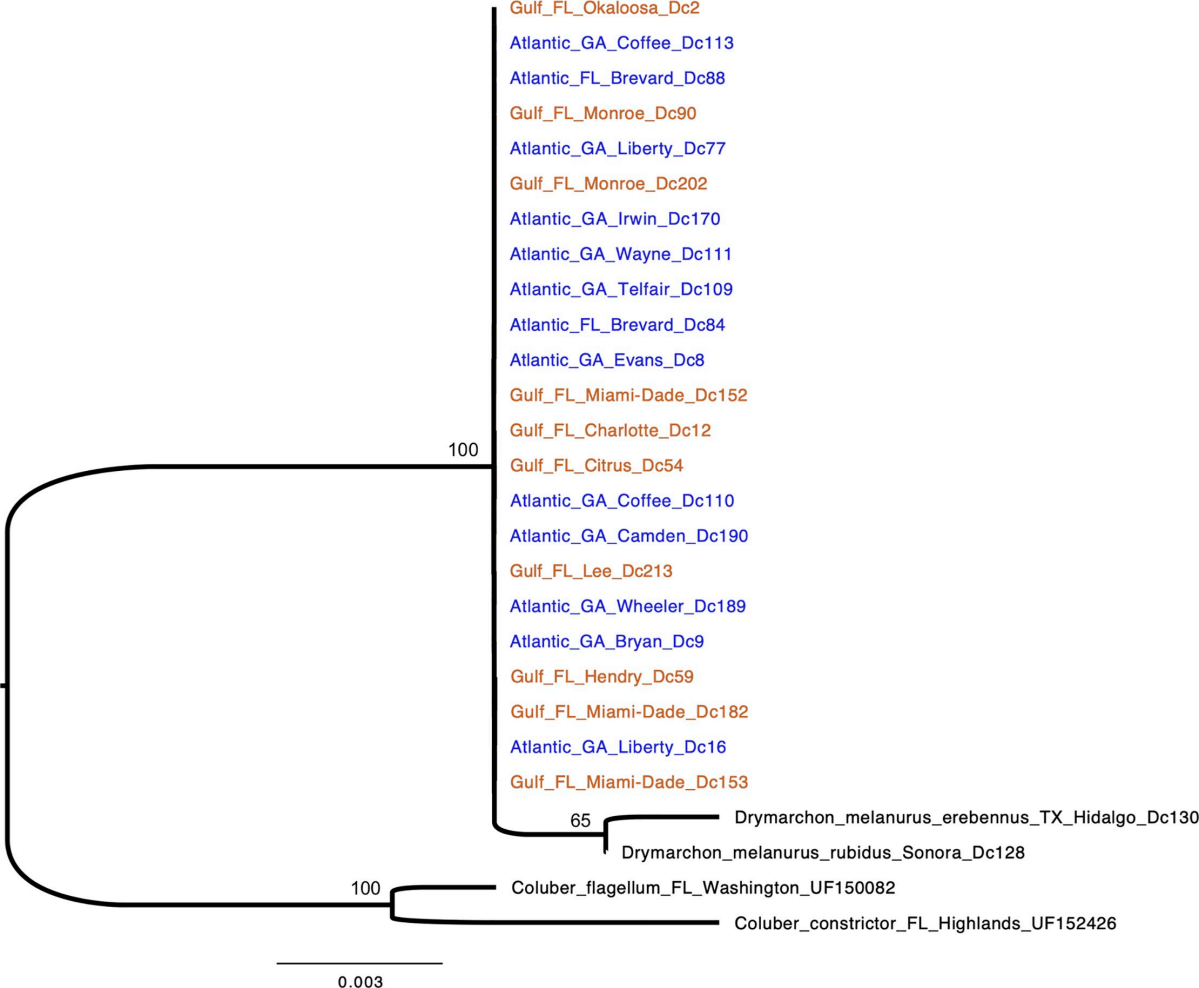
Maximum-likelihood phylogeny of Eastern Indigo Snakes (*Drymarchon couperi*) and outgroups inferred from sequence data from the nuclear gene *neurotrophin*-3 (NT3). Indigo snake samples are labeled by hypothetical lineage, state, county, and sample numbers from [26]. Colors: blue = Atlantic lineage, orange = Gulf lineage. Bootstrap support is listed for major nodes.

Model selection suggested that the partitioned mitochondrial data were best fit by the following models: HKY+F+I+G4 for the 1^st^ codon, TIM2+F+I for the 2^nd^ codon, TN+F+G4 for the 3^rd^ codon, and TIM2e (tRNAs); the nuclear locus was best fit by the Kimura [78] model. The mtDNA markers had 60 parsimony-informative sites among specimens of *D*. *couperi*, and the ML phylogeny from mtDNA recovered a Gulf lineage that rendered the Atlantic lineage paraphyletic (Fig 5). We found that the nuclear locus NT3 was completely invariant across all *D*. *couperi* specimens and only had parsimony-informative sites for phylogenetic inference of outgroups with respect to *D*. *couperi*. As expected, the ML phylogeny inferred from NT3 estimated a polytomy for *D*. *couperi* specimens (Fig 6), indicating a lack of phylogenetic structure among individuals from the two mitochondrial lineages from [26]. After 100 million generations, our BEAST MCMC chains failed to converge and produce ESS values greater than 200 for all parameters. This was true for under all the analysis settings we explored. As a result, we do not present the BEAST results.

## Discussion

Our analyses found evidence of genetic structure within populations of *D*. *couperi*, particularly along a north-south gradient and between southern Georgia and peninsular Florida; however, patterns of population genetic and phylogenetic structure revealed substantial differences from the phylogenetic structure used to generate the two-species hypothesis. First, the geographic pattern of genetic similarity suggested a north-south orientation of (1) three Georgia population clusters and five peninsular Florida population clusters (Structure), and (2) three Georgia population clusters, two south Florida clusters, and one wide-spread, peninsular-Florida cluster (Geneland). Both Structure and Geneland produced fairly consistent results, particularly with respect to samples from Georgia, but neither analysis supported the Gulf-Atlantic dichotomy predicted by the two-species hypothesis. Second, and most importantly for the two-species hypothesis, both Bayesian clustering analyses and the sPCA analyses documented widespread contemporary admixture of alleles among populations along the Florida peninsula. Admixture occurred across the entire range of *D*. *couperi* and cannot be characterized as a narrow hybrid zone. In fact, the greatest degree of admixture occurred across the contact zone between the two putative species with no evidence of genetic differentiation. While our MLPE analyses found support for isolation by distance both across all samples and within each mtDNA lineage, those analyses also found a high degree of overlap in the genetic distances within and between the mitochondrial lineages used to generate the two-species hypothesis. Further, our sPCA results, which also accounted for spatial autocorrelation among samples, were consistent with isolation by distance along a north-south gradient and are not consistent with the nearly-discrete distributions and low dispersal ascribed to the Gulf and Atlantic lineages by previous authors [26]. These results collectively indicate that genetic structure among populations is best described as continuous isolation by distance along the major north-south geographic axis within a single species.

The two-species hypothesis for *D*. *couperi* [16,26] was formed on the basis of evidence describing lineage separation between populations of *D*. *couperi*; however, our separate analyses of mitochondrial and nuclear gene sequence data failed to support this hypothesis. Our phylogenetic analysis of the nuclear locus NT3 recovered no support for the two-species hypothesis because this marker was identical for all individuals; instead, all phylogenetic structure of *D*. *couperi* inferred from sequence data was associated with the mitochondrial genome. Phylogenetic structure inferred from mtDNA recovered a monophyletic Gulf lineage that was nested within a paraphyletic Atlantic lineage, a result inconsistent with previously reported phylogenetic structure for *D*. *couperi* [16,26]. Because the two-species hypothesis requires strong lineage separation between populations and we were unable to recover phylogenetic structure consistent with that hypothesis using both nuclear and mitochondrial markers, we consider these results along with our population genetic results as sufficient to reject the two-species hypothesis. We therefore place *Drymarchon kolpobasileus* into synonomy with *D*. *couperi*.

Our study has broad implications for the conservation management of *D*. *couperi*. First, because *D*. *couperi* is federally-protected as Threatened under the Endangered Species Act [28,29], efforts to conserve and recover the species can again operate under the hypothesis that *D*. *couperi* is a single species. For example, *D*. *couperi* is now being repatriated to Conecuh National Forest, Alabama, and Apalachicola Bluffs and Ravines Preserve, Florida. Genetic stock from southeastern Georgia is primarily being used for these efforts; founder females used to generate captive stock for repatriation were sampled from Georgia and represent individuals from the three Georgia populations revealed by both Geneland and Structure. Our evaluation of population genetic structure indicates that no error of releasing the wrong historical entity (i.e., species) to repatriation sites is being made, as was argued by Krysko et al. [26]. Rather, additional life history information suggests the source populations provide appropriate stock for repatriation programs. Specifically, *D*. *couperi* in northern populations are thought to be dependent upon Gopher Tortoise (*Gopherus polyphemus*) burrows for winter refugia, likely because of milder winter temperatures experienced by populations at those latitudes [38–40,79,80]. Because the release sites are along the northern boundary of habitats historically occupied by *D*. *couperi*, Georgia source populations were chosen for repatriation stock to account for potential genetic components associated with refuge-seeking behavior and cold-climate physiology.

Our knowledge of *D*. *couperi* life history and spatial ecology allows us to generate hypotheses for the discordant patterns of phylogenetic and population genetic structure observed here. *Drymarchon couperi*, particularly males, have extremely large home ranges and great vagility [38,39]. Males can move up to ca. 2 km in a single day and have greater daily movement distances (up to two times greater) and home range sizes (2.5–6.6 times larger) than females [40]. Given the disparity of movement between males and females, we hypothesize that limited female movement may drive phylogenetic structure of the maternally-inherited mtDNA, while high levels of male movement drive extensive gene flow of the nuclear genome [81]. This life history-based model of intersexual variance in *D*. *couperi* gene flow is consistent with other reptile systems for which life-history strategies generate contrasting patterns of gene flow among populations. First, female philopatry of Loggerhead Sea Turtles (*Caretta caretta*) causes structuring of mtDNA in the Atlantic Ocean, but high male dispersal drives significant nuclear gene flow among populations [82]. Second, a recent study of Neotropical snakes found active-foraging species to have greater rates of nuclear gene flow than ambush-predator species with more limited dispersal [83]. Last, and most relevant, many squamates are polygynous and are characterized by greater male dispersal relative to females (S1 Table) [84–87]; therefore, squamates are expected to have greater gene flow of nDNA than mtDNA [81], a pattern is also seen in waterfowl [23,25]. Thus, we suggest that *D*. *couperi* is similar to other reptile species in that life history provides explanations for why phylogeographic patterns from mtDNA are predicted to be inconsistent with historical and/or contemporary patterns of nuclear DNA.

The North American Coastal Plain is a global biodiversity hotspot [88], and peninsular Florida has a high proportion of endemic species (e.g., snakes: [89–91]), which are likely a product of refugial isolation on islands during periods of elevated sea level in recent epochs and contemporary drainage-driven endemism [46,92]. While recent species delimitation exercises have sought to further delimit peninsular Florida populations as distinct species relative to their mainland counterparts [13,15,26,89–91], our examination of *D*. *couperi* adds to a growing number of examples of southeastern North American organisms that appear, based on modeling of one or a few genetic loci, to represent species that are distinct from other mainland counterparts, but for which microsatellite or similar data demonstrate substantial contemporary gene flow. Burbrink and Guiher [89] estimated that there was such low gene flow between cottonmouths (*Agkistrodon piscivorus*) in peninsular Florida and the mainland that speciation must have occurred between those two regions, a hypothesis immediately contested by data from Strickland et al. [93] who detected a broad geographic range of admixture using AFLP markers. Similarly, Thomas et al. [13] described alligator snapping turtles (*Macrochelys temminckii*) from the Apalachicola River and adjacent rivers to be a distinct species, despite microsatellite data from Echelle et al. [94] that are inconsistent with this conclusion [95]. While analyses of one or a few genetic loci can be informative by revealing apparent monophyly, they should not be viewed as sufficient to diagnose and delimit species, particularly when available life history data provide plausible mechanisms for contemporary gene flow and mito-nuclear discordance. In the case of *A*. *piscivorus*, the wetland habitats occupied by this species are abundant and extensive throughout the range of the species, which limits opportunity for population isolation within the core of the geographic range. *Agkistrodon piscivorus* are known to move among wetlands [96], providing ample opportunity for contemporary gene flow along peninsular Florida despite historical periods of isolation via marine inundation [46,92]. Similarly, observations of barnacles growing on shells of *M*. *temminckii* [97] indicate that this species can occupy brackish habitats, a life-history feature providing an avenue of dispersal between major drainages [94]. Thus, life history data exist for both species that demand examination of contemporary admixture and gene flow using multi-locus datasets, similar to our process with *D*. *couperi*. We point to papers that carefully meld phylogenetic and population genetic analyses [98–100] as examples of processes by which researchers might evaluate phylogenetic and population genetic datasets to delimit species. We argue that a more-consistent and robust voice from the community of systematists will emerge when life-history data are incorporated more strongly into the process of species delimitation [101]. When sex-biased dispersal is evident from ecological studies (e.g., squamates [102]), the utility of phylogenetic analyses dominated by the mitochondrial genome to reveal novel species should be questioned. We suggest that reviewers be particularly critical of species descriptions lacking analysis of population admixture and gene flow, because of the high costs of erroneous diversity on the conservation of imperiled biodiversity.

## Supporting information

S1 Fig Bar plots of population genetic clustering of *Drymarchon couperi* estimated through the Bayesian clustering algorithm Structure with (A) *K* = 6, (B) *K* = 7, (C) *K* = 9, and (D) *K* = 10. The y-axis is the proportion of individual ancestry for each cluster; in the x-axis, number represents county where sample was collected. County names for each number are shown in S5 Table. Counties 12–26 are overlapping on the x-axis due to small sample size per cluster. See Fig 1 for clustering analysis estimated at *K* = 8.
(TIFF)

S2 Fig Distributions of clusters along the entire MCMC chain and after burn-in from the Geneland independent run with the highest mean posterior, *K* = 6. (TIFF)

S3 Fig Results of spatial principle components analysis for population genetic data of *Drymarchon couperi* using a Delaunay triangulation connection network. The eigenvalues (left) for each axis where positive values indicate global structure and negative values indicate local structure, and the Moran’s *I* (right) plotted against the variance for each axis.
(TIFF)

S4 Fig Additional results of spatial principle components analysis for population genetic data of *Drymarchon couperi* using a Delaunay triangulation connection network demonstrating the spatially lagged scores from the first (A) and second (B) axes. Atlantic lineage samples are displayed using circles while Gulf lineage samples are displayed using squares. Samples with more extreme values/colors are more genetically differentiated.
(TIFF)

S5 Fig Results of spatial principle components analysis using a distance-based connection network where samples are considered connected if they are within 22.2 km which is the maximum recorded dispersal distance for *Drymarchon couperi*. The left figure shows the eigenvalues for each axis where positive values indicate global structure and negative values indicate local structure and the right figure shows the Moran’s *I* plotted against the variance for each axis.
(TIFF)

S1 Table Intersexual variance in home range size (hectares) for 22 species of snakes. See [103] for an earlier review of the topic.
(DOCX)

S2 Table Multiplex PCR panels for *Drymarchon couperi* microsatellite loci. The names of loci are as in [52].
(DOCX)

S3 Table GenBank accession numbers for gene sequence data (CytB, ND4, NT3) for *Drymarchon couperi* and outgroups from [26]. (DOCX)

S4 Table Estimated values of *K* for the four metrics proposed by Puechmaille [55]: Median of medians (MedMedK), median of means (MedMeanK), maximum of medians (MaxMedK), and maximum of means (MaxMeanK). Each metric is estimated at four different thresholds of cluster membership.
(DOCX)

S5 Table Numbers used to group samples by geographic states and counties for use in the Bayesian clustering algorithm Structure; see Fig 1. (DOCX)
