# Supplementary figures and images for: Taxonomic and conservation implications of population genetic admixture, mito-nuclear discordance, and male-biased dispersal of a large endangered snake, Drymarchon couperi
Source: PLoS One. 2019 Mar 26;14(3):e0214439. doi: 10.1371/journal.pone.0214439 (PMC6435180; doi:10.1371/journal.pone.0214439)

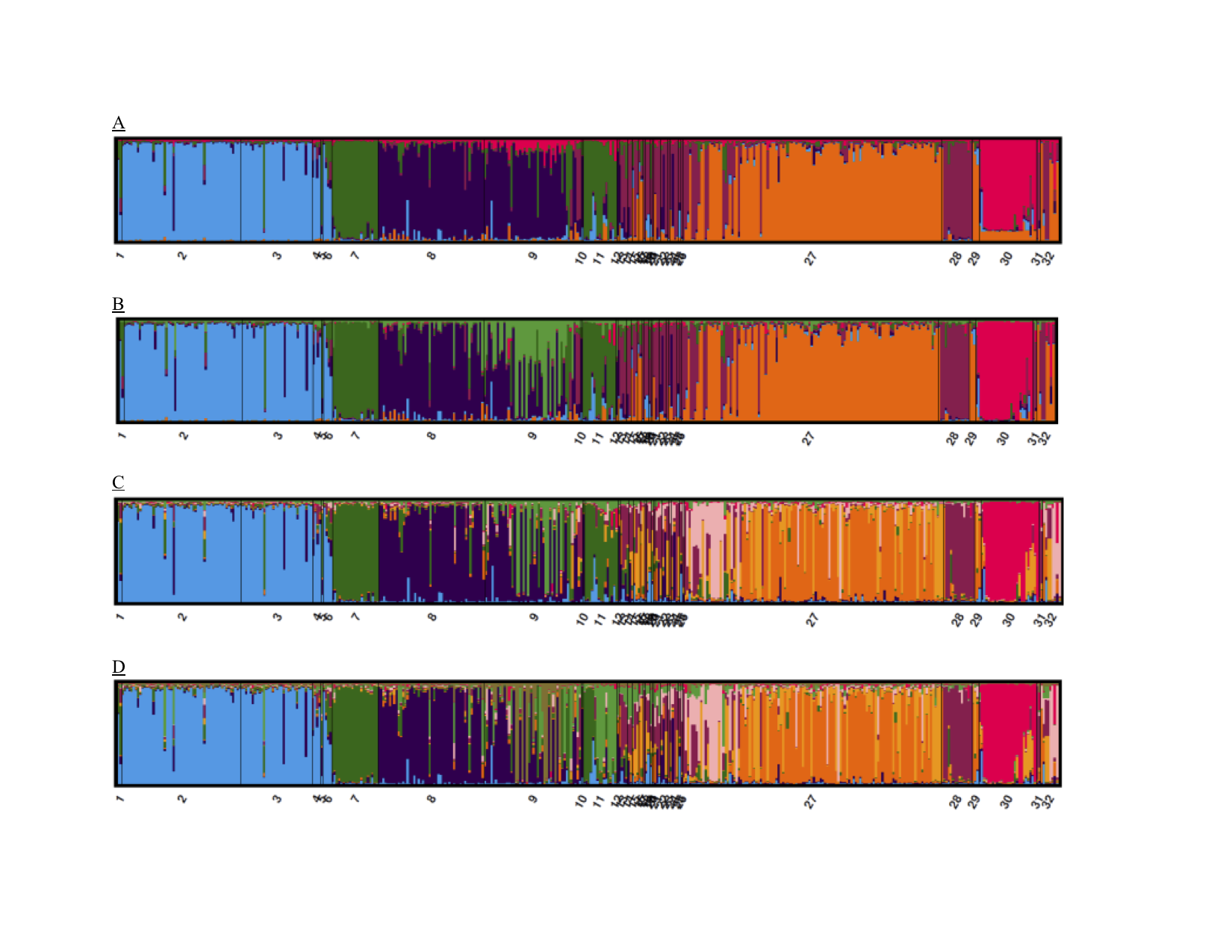

Supplement: S1 Fig — Bar plots of population genetic clustering of Drymarchon couperi estimated through the Bayesian clustering algorithm Structure with (A) K = 6, (B) K = 7, (C) K = 9, and (D) K = 10. The y-axis is the proportion of individual ancestry for each cluster; in the x-axis, number represents county where sample was collected. County names for each number are shown in S5 Table. Counties 12–26 are overlapping on the x-axis due to small sample size per cluster. See Fig 1 for clustering analysis estimated at K = 8. (TIFF) [file pone.0214439.s001.tiff]

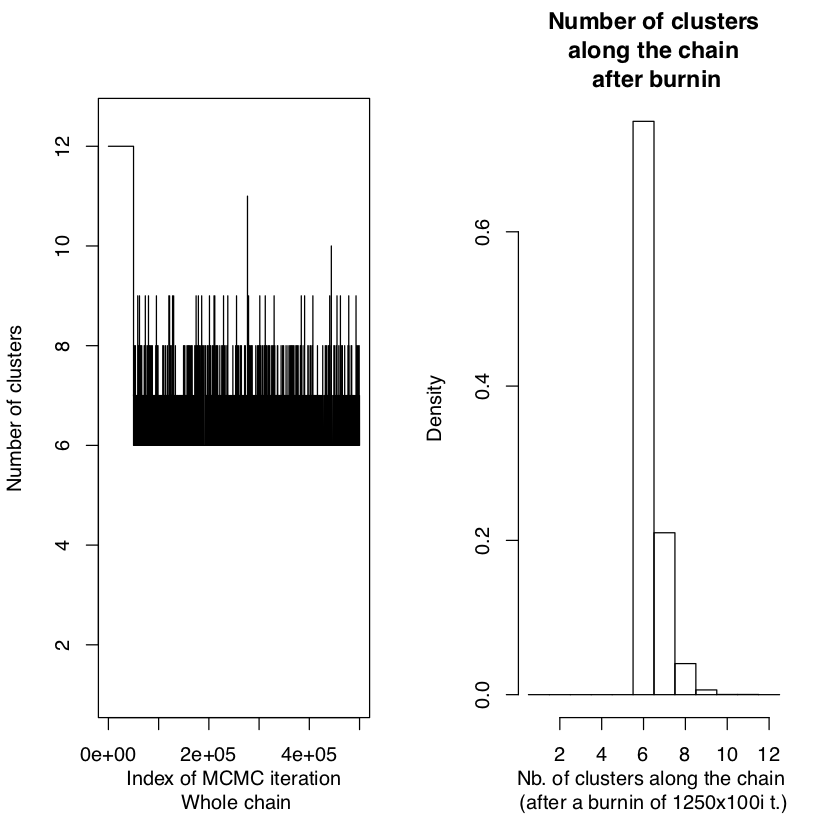

Supplement: S2 Fig — (TIFF) [file pone.0214439.s002.tiff]

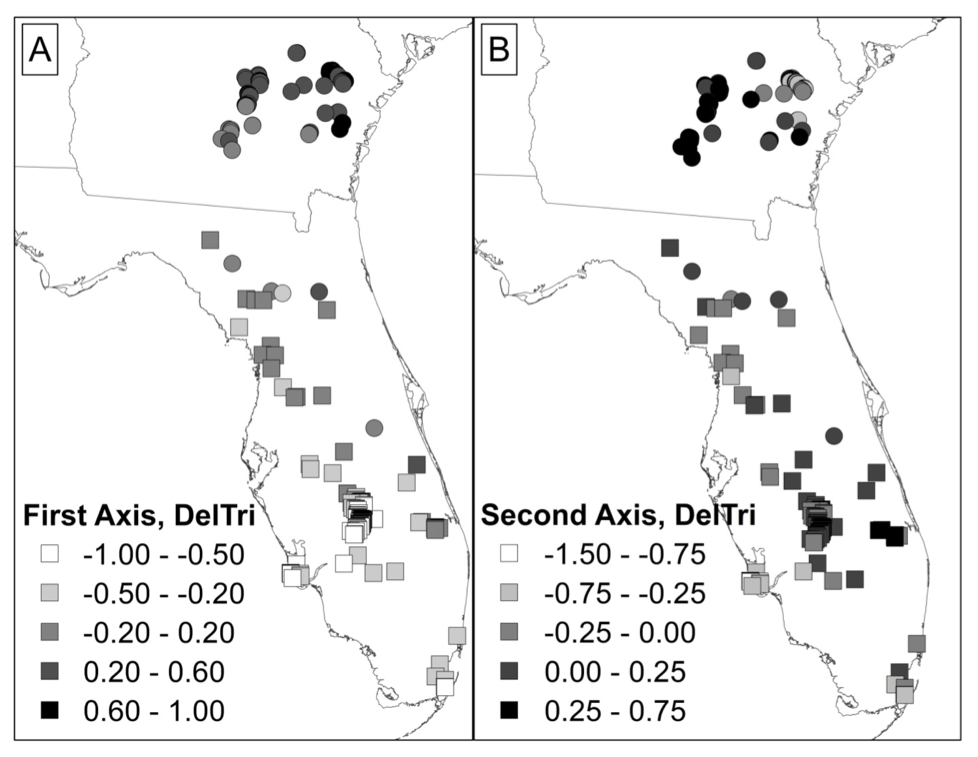

Supplement: S4 Fig — Additional results of spatial principle components analysis for population genetic data of Drymarchon couperi using a Delaunay triangulation connection network demonstrating the spatially lagged scores from the first (A) and second (B) axes. Atlantic lineage samples are displayed using circles while Gulf lineage samples are displayed using squares. Samples with more extreme values/colors are more genetically differentiated. (TIFF) [file pone.0214439.s004.tiff]

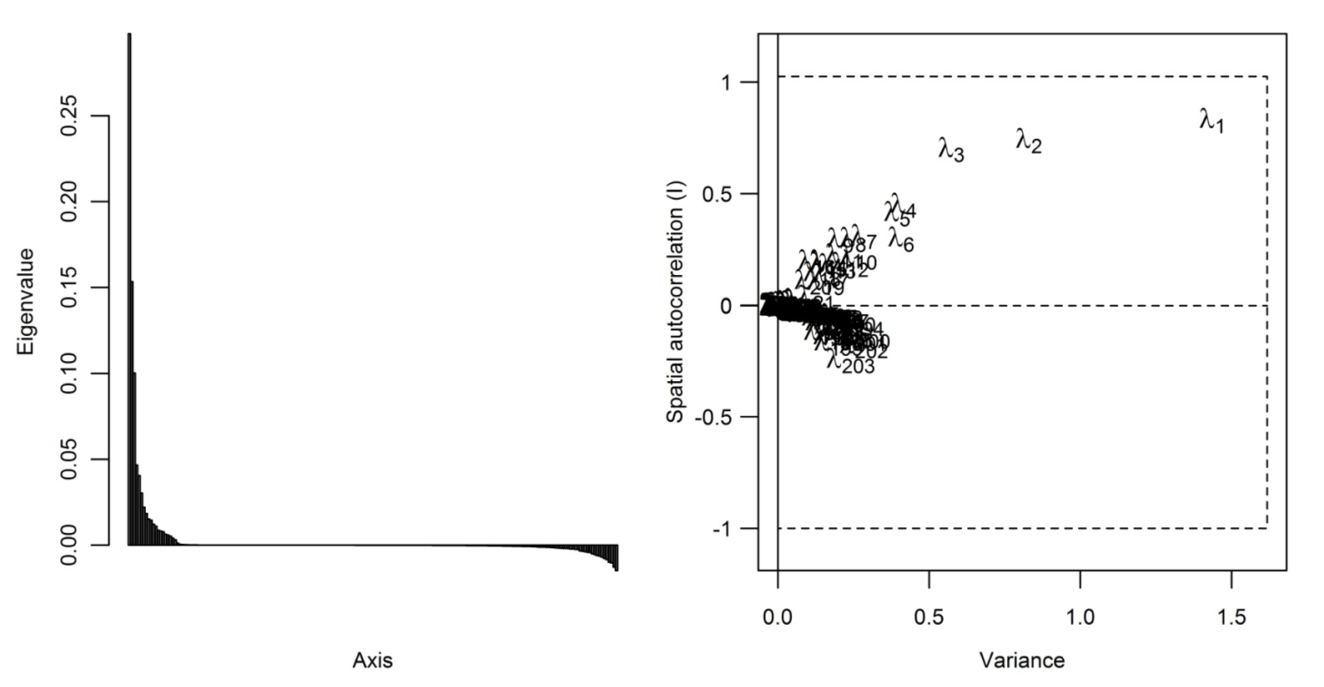

Supplement: S5 Fig — The left figure shows the eigenvalues for each axis where positive values indicate global structure and negative values indicate local structure and the right figure shows the Moran’s I plotted against the variance for each axis. (TIFF) [file pone.0214439.s005.tiff]
