# Supplementary material for: Taxonomic and conservation implications of population genetic admixture, mito-nuclear discordance, and male-biased dispersal of a large endangered snake, Drymarchon couperi
Source: PLoS One. 2019 Mar 26;14(3):e0214439. doi: 10.1371/journal.pone.0214439 (PMC6435180; doi:10.1371/journal.pone.0214439)
Supplement: S1 Table — See [103] for an earlier review of the topic. (DOCX) [file pone.0214439.s006.docx]

| Species | Home range size (ha) | | Citations |
| --- | --- | --- | --- |
|  | Female | Male |  |
| *Carphophis amoenus* | 0.004 | 0.028 | Clark (1970) |
| *Vipera aspera* | 0.18 | 0.29 | Naulleau (1968) |
| *Agkistrodon piscivorus* | 0.31 | 1.86 | Roth (2005) |
| *Coronella austriaca* | 0.45 | 0.66 | Goddard (1980) |
| *Nerodia sipedon* | 0.58 | 0.51 | Roth and Greene (2006) |
| *Pseudonaja textilis* | 1.5 | 11.8 | Whitaker and Shine (2003) |
| *Coluber constrictor* | 1.8 | 3 | Fitch (1963b) |
| *Hoplocephalus bungaroides* | 2.1 | 2.2 | Webb and Shine (1997) |
| *Agkistrodon contortrix* | 3.4 | 9.9 | Fitch (1960) |
| *C. constrictor* | 3.9 | 9.3 | Fitch (1958) |
| *Hoplocephalus stephensii* | 5.4 | 20.2 | Fitzgerald et al. (2002) |
| *Bothrops asper* | 5.8 | 6.3 | Wasko and Sasa (2009) |
| *Crotalus viridis* | 6.5 | 12.1 | Fitch (1949) |
| *Thamnophis sirtalis* | 9.2 | 14.2 | Fitch (1965) |
| *Pantherophis obsoletus* | 9.3 | 11.7 | Fitch (1963a) |
| *P. obsoletus* | 9.5 | 9.5 | Durner and Gates (1993) |
| *Morelia spilota* | 12 | 12 | Shine and Fitzgerald (1996) |
| *Drymarchon couperi* | 19 | 74 | Layne and Steiner (1996) |
| *Crotalus horridus* | 19.9 | 143.8 | Rudolph and Burgdof (1997) |
| *Coluber flagellum* | 21 | 150 | Dodd and Barichivich (2007) |
| *Sistrurus catenatus* | 21.7 | 27.8 | Johnson (2000) |
| *D. couperi* | 22 | 141 | Moler (1987) |
| *Pituophis catenifer* | 26.9 | 36.6 | Kapfer et al. (2010) |
| *Pituophis melanoleucus* | 37.5 | 70.1 | Miller et al. (2012) |
| *P. melanoleucus* | 43 | 69 | Gerald et al. (2006) |
| *D. couperi* | 49 | 149 | Bauder et al. (2016a) |
| *Lampropeltis getula* | 49.4 | 49.5 | Linehan et al. (2010) |
| *D. couperi* | 76 | 202 | Breininger et al. (2011) |
| *D. couperi* | 101 | 510 | Hyslop (2007) |
|  |  |  |  |
