# Supplementary material for: Taxonomic and conservation implications of population genetic admixture, mito-nuclear discordance, and male-biased dispersal of a large endangered snake, Drymarchon couperi
Source: PLoS One. 2019 Mar 26;14(3):e0214439. doi: 10.1371/journal.pone.0214439 (PMC6435180; doi:10.1371/journal.pone.0214439)
Supplement: S2 Table — The names of loci are as in [52]. (DOCX) [file pone.0214439.s007.docx]

| Multiplex | Locus |
| --- | --- |
| 1 | Dry24 – 6FAM |
|  | Dry55 – 6FAM |
|  | Dry30 – VIC |
|  | Dry44 – PET |
|  | Dry68 - NED |
|  |  |
| 2 | Dry48 – 6FAM |
|  | Dry63 – 6FAM |
|  | Dry58 – VIC |
|  | Dry59 – VIC |
|  | Dry65 – VIC |
|  | Dry69 - VIC |
|  | Dry06 – PET |
|  | Dry33 - NED |
|  |  |
| 3 | Dry05 – 6FAM |
|  | Dry35 – VIC |
|  | Dry14 – PET |
|  | Dry70 – NED |
|  |  |
