# Supplementary material for: Taxonomic and conservation implications of population genetic admixture, mito-nuclear discordance, and male-biased dispersal of a large endangered snake, Drymarchon couperi
Source: PLoS One. 2019 Mar 26;14(3):e0214439. doi: 10.1371/journal.pone.0214439 (PMC6435180; doi:10.1371/journal.pone.0214439)
Supplement: S3 Table — (DOCX) [file pone.0214439.s008.docx]

| Species | Krysko et al. lineage | Country | State | County | Sample Number | CytB | ND4 | NT3 |
| --- | --- | --- | --- | --- | --- | --- | --- | --- |
| *Drymarchon couperi* | Gulf | USA | FL | Charlotte | Dc12 | KM386579 | KP099145 | KT214324 |
| *Drymarchon couperi* | Gulf | USA | FL | Citrus | Dc76 | KM386573 | KP099139 |  |
| *Drymarchon couperi* | Gulf | USA | FL | Highlands | Dc97 | KM386578 | KP099144 |  |
| *Drymarchon couperi* | Gulf | USA | FL | Marion | Dc19 | KM386548 | KP099115 |  |
| *Drymarchon couperi* | Gulf | USA | FL | Okaloosa | Dc137 | KM386605 | KP099171 |  |
| *Drymarchon couperi* | Gulf | USA | FL | Marion | Dc56 | KM386610 | KP099176 |  |
| *Drymarchon couperi* | Gulf | USA | FL | Okaloosa | Dc2 | KM386597 | KP099163 | KT214325 |
| *Drymarchon couperi* | Gulf | USA | FL | Gilchrist | Dc200 | KM386556 | KP099123 |  |
| *Drymarchon couperi* | Gulf | USA | FL | Hernando | Dc185 | KM386594 | KP099160 |  |
| *Drymarchon couperi* | Gulf | USA | FL | Hernando | Dc186 | KM386598 | KP099164 |  |
| *Drymarchon couperi* | Gulf | USA | FL | Hillsborough | Dc160 | KM386593 | KP099159 |  |
| *Drymarchon couperi* | Gulf | USA | FL | Lake | Dc150 | KM386582 | KP099148 |  |
| *Drymarchon couperi* | Gulf | USA | FL | Lee | Dc213 | KT447211 | KT447211 | KT214326 |
| *Drymarchon couperi* | Gulf | USA | FL | Lake | Dc4 | KM386559 | KP099126 |  |
| *Drymarchon couperi* | Gulf | USA | FL | Levy | Dc5 | KM386570 | KP099136 |  |
| *Drymarchon couperi* | Gulf | USA | FL | Marion | Dc15 | KM386581 | KP099147 |  |
| *Drymarchon couperi* | Gulf | USA | FL | Pasco | Dc13 | KM386554 | KP099121 |  |
| *Drymarchon couperi* | Gulf | USA | FL | Indian River | Dc146 | KM386571 | KP099137 |  |
| *Drymarchon couperi* | Gulf | USA | FL | Alachua | Dc10 | KM386580 | KP099146 |  |
| *Drymarchon couperi* | Gulf | USA | FL | Alachua | Dc11 | KM386600 | KP099166 |  |
| *Drymarchon couperi* | Gulf | USA | FL | Citrus | Dc54 | KM386592 | KP099158 | KT214327 |
| *Drymarchon couperi* | Gulf | USA | FL | Citrus | Dc60 | KM386608 | KP099174 |  |
| *Drymarchon couperi* | Gulf | USA | FL | Marion | Dc55 | KM386583 | KP099149 |  |
| *Drymarchon couperi* | Gulf | USA | FL | Collier | Dc148 | KM386565 | KP099131 |  |
| *Drymarchon couperi* | Gulf | USA | FL | Hendry | Dc184 | KM386551 | KP099118 |  |
| *Drymarchon couperi* | Gulf | USA | FL | Hendry | Dc59 | KM386549 | KP099116 | KT214328 |
| *Drymarchon couperi* | Gulf | USA | FL | Miami-Dade | Dc192 | KM386586 | KP099152 |  |
| *Drymarchon couperi* | Gulf | USA | FL | Monroe | Dc90 | KM386596 | KP099162 | KT447213 |
| *Drymarchon couperi* | Gulf | USA | FL | Hendry | Dc91 | KM386611 | KP099177 |  |
| *Drymarchon couperi* | Gulf | USA | FL | Highlands | Dc96 | KM386563 | KP099129 |  |
| *Drymarchon couperi* | Gulf | USA | FL | Miami-Dade | Dc182 | KM386546 | KP099113 | KT214329 |
| *Drymarchon couperi* | Gulf | USA | FL | Miami-Dade | Dc193 | KM386557 | KP099124 |  |
| *Drymarchon couperi* | Gulf | USA | FL | Palm Beach | Dc127 | KM386552 | KP099119 |  |
| *Drymarchon couperi* | Gulf | USA | FL | Highlands | Dc131 | KM386577 | KP099143 |  |
| *Drymarchon couperi* | Gulf | USA | FL | Okeechobee | Dc144 | KM386547 | KP099114 |  |
| *Drymarchon couperi* | Gulf | USA | FL | Polk | Dc138 | KM386612 | KP099178 |  |
| *Drymarchon couperi* | Gulf | USA | FL | Highlands | Dc103 | KM386575 | KP099141 |  |
| *Drymarchon couperi* | Gulf | USA | FL | Highlands | Dc132 | KM386606 | KP099172 |  |
| *Drymarchon couperi* | Gulf | USA | FL | Hendry | Dc133 | KM386574 | KP099140 |  |
| *Drymarchon couperi* | Gulf | USA | FL | Miami-Dade | Dc152 | KM386550 | KP099117 | KT214330 |
| *Drymarchon couperi* | Gulf | USA | FL | Miami-Dade | Dc153 | KM386601 | KP099167 | KT214331 |
| *Drymarchon couperi* | Gulf | USA | FL | Highlands | Dc159 | KM386576 | KP099142 |  |
| *Drymarchon couperi* | Gulf | USA | FL | Martin | Dc194 | KM386590 | KP099156 |  |
| *Drymarchon couperi* | Gulf | USA | FL | Monroe | Dc202 | KM386568 | KP099134 | KT214332 |
| *Drymarchon couperi* | Atlantic | USA | FL | Alachua | Dc58 | KM386614 | KP099180 |  |
| *Drymarchon couperi* | Atlantic | USA | GA | Coffee | Dc113 | KM386616 | KP099182 | KT214333 |
| *Drymarchon couperi* | Atlantic | USA | GA | Liberty | Dc77 | KM386609 | KP099175 | KT214334 |
| *Drymarchon couperi* | Atlantic | USA | GA | Wayne | Dc111 | KM386603 | KP099169 | KT214335 |
| *Drymarchon couperi* | Atlantic | USA | GA | Atkinson | Dc197 | KM386572 | KP099138 |  |
| *Drymarchon couperi* | Atlantic | USA | FL | Nassau | Dc124 | KM386566 | KP099132 |  |
| *Drymarchon couperi* | Atlantic | USA | GA | Atkinson | Dc210 | KM386589 | KP099155 |  |
| *Drymarchon couperi* | Atlantic | USA | GA | Berrien | Dc209 | KM386613 | KP099179 |  |
| *Drymarchon couperi* | Atlantic | USA | GA | Bryan | Dc9 | KM386587 | KP099153 | KT214336 |
| *Drymarchon couperi* | Atlantic | USA | GA | Camden | Dc190 | KM386595 | KP099161 | KT214337 |
| *Drymarchon couperi* | Atlantic | USA | GA | Coffee | Dc110 | KM386545 | KP099112 | KT214338 |
| *Drymarchon couperi* | Atlantic | USA | GA | Coffee | Dc198 | KM386569 | KP099135 |  |
| *Drymarchon couperi* | Atlantic | USA | GA | Telfair | Dc109 | KM386585 | KP099151 | KT214339 |
| *Drymarchon couperi* | Atlantic | USA | GA | Evans | Dc8 | KM386607 | KP099173 | KT214340 |
| *Drymarchon couperi* | Atlantic | USA | GA | Irwin | Dc170 | KM386567 | KP099133 | KT214341 |
| *Drymarchon couperi* | Atlantic | USA | GA | Wheeler | Dc189 | KM386558 | KP099125 | KT447212 |
| *Drymarchon couperi* | Atlantic | USA | FL | Alachua | Dc14 | KM386584 | KP099150 |  |
| *Drymarchon couperi* | Atlantic | USA | GA | Liberty | Dc16 | KM386604 | KP099170 | KT214342 |
| *Drymarchon couperi* | Atlantic | USA | FL | Brevard | Dc81 | KM386602 | KP099168 |  |
| *Drymarchon couperi* | Atlantic | USA | FL | Brevard | Dc84 | KM386588 | KP099154 | KT214343 |
| *Drymarchon couperi* | Atlantic | USA | FL | Brevard | Dc88 | KM386564 | KP099130 | KT214344 |
| *Drymarchon couperi* | Atlantic | USA | FL | Putnam | Dc164 | KM386562 | KP099128 |  |
| *Drymarchon couperi* | Atlantic | USA | FL | Osceola | Dc188 | KM386617 | KP099183 |  |
| *Drymarchon couperi* | Atlantic | USA | FL | Osceola | Dc129 | KM386544 | KP099111 |  |
| *Drymarchon couperi* | Atlantic | USA | GA | Charlton | Dc191 | KM386553 | KP099120 |  |
| *Drymarchon couperi* | Atlantic | USA | FL | Suwanee | Dc187 | KM386561 | KP099127 |  |
| *Drymarchon couperi* | Atlantic | USA | FL | Hamilton | Dc201 | KM386591 | KP099157 |  |
| *Drymarchon couperi* | Atlantic | USA | FL | St Johns | Dc69 | KM386615 | KP099181 |  |
| *Drymarchon melanurus erebennus* | NA | USA | TX | Hidalgo | Dc130 | KM386599 | KP099165 | KT214322 |
| *Drymarchon melanurus rubidus* | NA | MX | Sonora |  | Dc128 | KM386555 | KP099122 | KT214323 |
| *Masticophis flagellum* | NA | USA | FL | Washington | UF150082 | KM403637 | KT447217 | KT447214 |
| *Coluber constrictor* | NA | USA | FL | Highlands | UF152426 | KM386560 | KT447216 | KT447215 |
| *Salvadora mexicanum* | NA | MX | Michoacan | |  | AY486934 | AY487075 |  |
|  |  |  |  |  |  |  |  |  |
