# Supplementary material for: Taxonomic and conservation implications of population genetic admixture, mito-nuclear discordance, and male-biased dispersal of a large endangered snake, Drymarchon couperi
Source: PLoS One. 2019 Mar 26;14(3):e0214439. doi: 10.1371/journal.pone.0214439 (PMC6435180; doi:10.1371/journal.pone.0214439)
Supplement: S4 Table — Each metric is estimated at four different thresholds of cluster membership. (DOCX) [file pone.0214439.s009.docx]

| Threshold | MedMedK | MedMeanK | MaxMedK | MaxMeanK |
| --- | --- | --- | --- | --- |
| 0.5 | 9 | 8 | 10 | 9 |
| 0.6 | 8 | 7 | 10 | 9 |
| 0.7 | 7 | 6 | 9 | 7 |
| 0.8 | 6 | 6 | 8 | 7 |
|  |  |  |  |  |
