# Supplementary material for: Taxonomic and conservation implications of population genetic admixture, mito-nuclear discordance, and male-biased dispersal of a large endangered snake, Drymarchon couperi
Source: PLoS One. 2019 Mar 26;14(3):e0214439. doi: 10.1371/journal.pone.0214439 (PMC6435180; doi:10.1371/journal.pone.0214439)
Supplement: S5 Table — (DOCX) [file pone.0214439.s010.docx]

| Number | State | County |
| --- | --- | --- |
| 1 | Georgia | Candler |
| 2 | Georgia | Bryan |
| 3 | Georgia | Evans |
| 4 | Georgia | Liberty |
| 5 | Georgia | Tattnall |
| 6 | Georgia | Long |
| 7 | Georgia | Wheeler |
| 8 | Georgia | Telfair |
| 9 | Georgia | Coffee |
| 10 | Georgia | Appling |
| 11 | Georgia | Wayne |
| 12 | Georgia | Berrien |
| 13 | Georgia | Atkinson |
| 14 | Georgia | Lafayette |
| 15 | Florida | Alachua |
| 16 | Georgia | Putnam |
| 17 | Florida | Osceola |
| 18 | Florida | Gilchrist |
| 19 | Florida | Levy |
| 20 | Florida | Marion |
| 21 | Florida | Citrus |
| 22 | Florida | Hernando |
| 23 | Florida | Lake |
| 24 | Florida | Polk |
| 25 | Florida | Indian River |
| 26 | Florida | Okeechobee |
| 27 | Florida | Highlands |
| 28 | Florida | Martin |
| 29 | Florida | Glades |
| 30 | Florida | Lee |
| 31 | Florida | Hendry |
| 32 | Florida | Miami-Dade |
